# *Drosophila melanogaster* larvae are tolerant to oral infection with the bacterial pathogen *Photorhabdus luminescens*

**DOI:** 10.17912/micropub.biology.000938

**Published:** 2023-08-29

**Authors:** Dhaivat Raval, Lillia Daley, Ioannis Eleftherianos

**Affiliations:** 1 Department of Biological Sciences, The George Washington University, Washington, DC, USA

## Abstract

The fruit fly *Drosophila melanogaster* is an excellent model for dissecting the molecular and functional bases of bacterial pathogenicity and host antibacterial immune response. The Gram-negative bacterium *Photorhabdus luminescens* is an insect-specific pathogen that forms a mutualistic relationship with the entomopathogenic nematode *Heterorhabditis bacteriophora* . Here we find that oral infection of *D. melanogaster* larvae with *P. luminescens* moderately reduces their survival ability while the bacteria replicate efficiently in the infected insects. This information will contribute towards understanding host gut immunity against potent bacterial pathogens.

**Figure 1. Drosophila melanogaster larval survival and bacterial load following oral infection with non-pathogenic and pathogenic bacteria:**
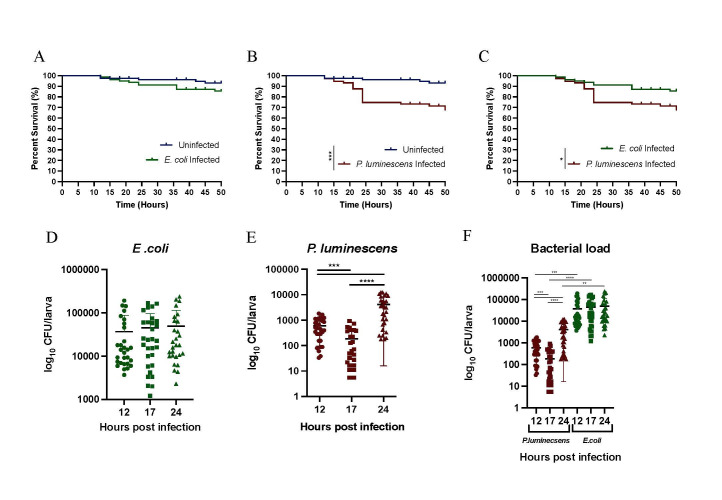
Survival percentage of *D. melanogaster* wild type Oregon-R larvae over a course of 50 hours following oral infection with (A) the non-pathogenic *Escherichia coli* strain K-12 or (B) the entomopathogenic bacterium *P. luminescens* TT01. Uninfected individuals kept on a sucrose supplemented with PBS were used as negative controls. (C) Comparison of survival curves between *D. melanogaster* wild type Oregon-R larvae following oral infection with either *E. coli* K-12 or *P. luminescens* TT01. Larval survival experiments were repeated three times, and eachexperiment involved at least 15 larvae. A Log-rank test was used to calculate statistically significant differences between the survival curves (*** P=0.0001, * P=0.0142). Log10 bacterial Colony Forming Units (CFU) per *D. melanogaster* wild type Oregon-R larva at 12, 17 and 24 hours following oral infection with (D) *E. coli* K-12 or (E) *P. luminescens* TT01. (F) Comparison of bacterial CFUs per *D. melanogaster* wild type Oregon-R larva at 12, 17 and 24 hours post oral infection with either *P. luminescens* TT01 or *E. coli* K-12. Bacterial load experiments were repeated three times, and eachexperiment involved 10 larvae per condition and two technical duplicates for each bacterial treatment. Welch’s t-test was used to calculate statistically significant differences between time points and bacterial treatments (**** P=0.0006, *** P<0.0001, ** P=0.011).

## Description

The fruit fly *Drosophila melanogaster* is an established model organism that is used extensively in host-pathogen interactions to understand key biological processes due to its evolutionarily conserved signaling pathways and transcriptional regulators (Harnish *et al* ., 2021). *Drosophila melanogaster* thrives in diverse natural environments containing a variety of microorganisms, many of which present immune challenges to its organ systems and especially the gut (Kuraishi *et al* ., 2013). The fly gut has physiological similarities to the mammalian gut and has evolved conserved mechanisms to withstand microbial infections (Miguel-Aliaga *et al* ., 2018; Capo *et al* ., 2019).

*Photorhabdus luminescens* is a bioluminescent Gram-negative bacterium that lives in a mutualistic association with species of the *Heterorhabditidae* nematode family (Abd-Elgawad, 2021) . During infection, once the host insect hemocoel is breached, the nematodes release the bacteria from their gut (Waterfield *et al* ., 2009; Clarke, 2020). The bacteria multiply rapidly while secreting toxins and virulence factors that damage vital insect tissues and weaken the insect immune system (Eleftherianos *et al* ., 2010). *Photorhabdus luminescens* can be successfully cultured away from its natural nematode host and is particularly pathogenic when injected directly into the *D. melanogaster* hemocoel (Aymeric *et al* ., 2010; Castillo *et al* ., 2012). However, the oral pathogenicity of *P. luminescens* toward *D. melanogaster* larvae has not been previously investigated in great detail.

Certain bacteria confer oral pathogenicity to *D. melanogaster* (Valet-Gely *et al* ., 2008; Buchon *et al* ., 2013). For example, when orally infected with wild type *Pseudomonas entomophila, D. melanogaster* adult flies have reduced survival compared to oral infection with a *P. entomophila* strain mutant for the toxic secretion factor GacA (Liehl *et al* ., 2006). Oral infection with the entomopathogenic bacterium *Xenorhabdus nematophila* significantly reduces survival of *D. melanogaster* larvae compared to infection with non-pathogenic bacteria (Peña *et al* ., 2015). Oral infection of *D. melanogaster* larvae with the Gram-negative pathogen *Psuedomonas fluorescens* leads to lethal and non-lethal effects. The non-lethal effects of *P. fluorescens* include reduction in body size, loss of fat body integrity, and prolonged larval development (Olcott *et al* ., 2010). Oral infection of *D. melanogaster* adults with the zoonotic pathogen *Serratia marcescens* promotes bacterial persistence in the hemolymph and increases fly sensitivity (Nehme et al., 2007) . Ingestion of the human pathogen *Vibrio cholerae* by *D. melanogaster* adults confers lethal effects to the flies and increases bacterial replication and persistence (Blow *et al* ., 2005). Interestingly, ingestion of the phytopathogen *Erwinia carotovora carotovora* 15 ( *Ecc* 15) by *D. melanogaster* larvae leads to immune activation without significantly affecting larval survival (Basset *et al* ., 2000).

Analysis of the survival response of *D. melanogaster* larvae to oral bacterial infection showed no statistically significant differences between the *E. coli* infected individuals compared to the uninfected controls ( Figure 1A ). However, statistically significant differences in survival were found between larvae orally infected with *P. luminescens* and uninfected individuals ( Figure 1B ), and those orally infected with *E. coli* ( Figure 1C ). These results indicated that *D. melanogaster* larvae infected with a non-pathogenic strain of *E. coli* had similar survival trend compared to uninfected individuals, whereas larvae infected with pathogenic *P. luminescens* had decreased survival compared to the other treatment groups.

For bacterial load estimation, larvae were collected, homogenized, and plated on selective and differentiating agar plates at 12, 17 and 24 hours following oral bacterial infection. The time points for bacterial load estimation were selected based on the survival curves. Analysis of the bacterial load over time in *D. melanogaster* larvae following *E. coli* ingestion revealed no statistically significant differences ( Figure 1D ). When orally infected with *P. luminescens* , there was a significantly higher number of bacteria in larvae at 12 and 24 hours compared to 17 hours post infection ( Figure 1E ), but a significantly lower bacterial load at 12 hours when compared to the 24-hour time point ( Figure 1E ). We also examined the *D. melanogaster* larval bacterial load following ingestion of *E. coli* or *P. luminescens* at the three selected time points. We found that the number of *Escherichia coli* cells in the infected wild type larvae was significantly higher compared to the number of *P. luminescens* cells for each of the time points ( Figure 1F ).

The results from the current survival experiments demonstrate that *P. luminescens* confers low to moderate oral pathogenicity to *D. melanogaster* wild type larvae. Previous research originally showed lack of *D. melanogaster* larval susceptibility to *P. luminescens* ingestion (Hallem *et al* ., 2007). However, subsequent work using GFP-labelled *P. luminescens* reported successful infection of fly larvae and up to 50% mortality after 72 hours of exposure to the pathogen. Also, another study found approximately 30% mortality of *D. melanogaster* wild type larvae responding to *P. luminescens* oral infection (Aymeric *et al* ., 2010). Although the survival results obtained in the present work are similar to the larval survival phenotypes in the latter study, it is important to emphasize that our experiments involved larvae of the *D. melanogaster* Oregon-R line, whereas the previous investigations used Canton-S and Cn bw larvae. We have previously documented strong variation in the immune response of *D. melanogaster* wild type adults against bacterial infection, and perhaps immune system variation also exists in the larval stage (Eleftherianos *et al* ., 2014). In addition, variation in survival rates between across different studies could be attributed to the infection method, given that *D. melanogaster* larvae can ingest *P. luminescens* through a sucrose solution or by exposing them to bacterial lawns, as well as to the number of bacterial cells used for infection.

Interestingly, here we find that oral *P. luminescens* infection confers moderate pathogenic effects on the larval host decreasing its survival, while oral *E. coli* infection has no detrimental effects on larval survival, despite the higher *E. coli* load compared to the lower *P. luminescens* burden in all three time points. The results of the current bacterial load experiments from *D. melanogaster* larvae fed on *E. coli* are consistent with the survival analysis, as *E. coli* bacteria have been previously shown to persist in the gut without altering larval survival, growth, or development (Van den Bergh 2022) . The results from the bacterial load analysis following exposure to *P. luminescens* display a decrease in pathogen Colony Forming Units (CFUs) at 17 hours, followed by a drastic increase at 24 hours. This is not due to a decreased larval feeding rate upon exposure to *P. luminescens* (our unpublished data). This pattern may be indicative of an interaction between the *D. melanogaster* larval immune response and the pathogen as the immune system unsuccessfully attempts to clear the infection. Alternatively, the reduced *P. luminescens* CFUs compared to *E. coli* could represent a strategy of the pathogen to restrain its own replication in the gut in order to interfere with host detection and consequent activation of the intestine local immune response. Of note, oral infection with *P. luminescens* elicits antimicrobial peptide gene transcription in *D. melanogaster* larvae, but not in adult flies (ffrench-Constant *et al* ., 2007; Castillo *et al* ., 2013). Whether this immune gene transcription pattern is specific to the gut remains to be investigated.

Future work will focus on the identification of the number and nature of genes which are differentially regulated in the *D. melanogaster* wild type larval gut during *P. luminescens* ingestion. Characterization of the transcriptomic profile in the gut, combined with the use of *D. melanogaster* larvae with mutations in candidate genes, will allow us to define the signaling pathways and molecular components responsible for the observed phenotypes. In addition, manipulation of the microbiome in the *D. melanogaster* larval gut will contribute toward a better understanding of the types of gut bacteria that participate in inter- and intra-species interactions in the context of infection with a potent entomopathogen such as *P. luminescens* (Broderick and Lemaitre, 2012; Trinder *et al* ., 2017). Considering that innate immune responses are highly conserved through evolution (Yu *et al* ., 2022), similar research will potentially reveal novel host factors that play a central role in modulating the intestinal immune defense against invading pathogens in humans.

## Methods

**Fly stocks.** Fly stocks were maintained on *D. melanogaster* medium-B and supplemented with baker’s yeast. The flies were kept at 25°C and on a 12:12 hour light dark photoperiod cycle. Late second to early third instar stage larvae of the *D. melanogaster* line Oregon-R (Bloomington, stock 5) were used throughout this study.

**Bacterial stocks.** *Photorhabdus luminescens,* subspecies *laumondii* , TT01 strain was cultured on MacConkey Agar (Sigma) at 30°C for 48 hours after which a single colony was selected and inoculated for 24 hours in 10 mL of liquid Lysogeny Broth (LB) media at 30°C on a shaker set at 210 rpm. *Escherichia coli,* strain K-12 was used as a bacterial control. It was cultured similarly at 37°C with the exception that the initial growth was performed on LB agar instead of MacConkey Agar.

**Larval survival.** *Drosophila melanogaster* larvae were starved for two hours and then infected with *E. coli* K-12 or *P. luminescens* TT01 in 96-well plates. Each well contained 100 µL of 1.25% agarose with 1x Phosphate Buffered Saline (PBS). The bacteria were suspended in a 1x PBS + 10% sucrose solution and reached an Optical Density between 25.0-26.5 at 600 nm. The bacterial suspension was added first to the well followed by the transfer of a single larva using a paint brush. Treatment of larvae with sucrose solution (10% in sterile 1xPBS) was used as negative control. The microtiter plate was covered with sealing film and two holes in each well were made for ventilation. All wells were replenished with sterile 1x PBS containing 10% sucrose solution every 24 hours. The plates were kept in the dark at 20-23°C and larval survival was estimated up to 50 hours post infection. Larval survival experiments were repeated three times, each with at least 15 biological replicates.

**Bacterial load estimation.** Following bacterial infection, *D. melanogaster* late second to early third instar stage larvae were collected at 12, 17 and 24-hours. Ten larvae per treatment were collected and homogenized using 70-100 µL of 1 mm glass beads in PCR tubes with 100 µL of sterile 1x PBS. The homogenates were serially diluted five times with sterile 1x PBS (1:11 ratio) in a 96-well plate using a multi-channel pipette. The diluted samples (100 µL) from *P. luminescens* infected larvae were spread on MacConkey agar and the diluted samples (100 µL) from *E. coli* infected larvae were spread on Harlequin agar (Neogen) using a sterile L- spreader. Two technical replicates were serially diluted and plated for each bacterial treatment. Two sets of one dilution (1:11 ratio) from uninfected larval homogenates were prepared. One set was spread on MacConkey agar and the other set was spread on Harlequin agar. Samples spread on MacConkey agar were incubated for 48 hours at 30°C and samples spread on Harlequin agar were incubated for 18-24 hours at 37°C. Following bacterial incubation, the agar plates were placed on a colony counter with a back light and the number of colonies was counted.

**Statistical Analysis.** Kaplan Meier curves were generated from the larval survival data and a log rank test was performed using GraphPad Prism 9 software. To calculate the CFUs in each sample, the serial dilutions of the colony counts were multiplied by their specific dilution factor. The CFU counts were plotted using GraphPad Prism 9, which was also used to carry out a two-tailed t test to analyze statistical significance differences between treatments.
